# A Height Estimation Approach for Terrain Following Flights from Monocular Vision

**DOI:** 10.3390/s16122071

**Published:** 2016-12-06

**Authors:** Igor S. G. Campos, Erickson R. Nascimento, Gustavo M. Freitas, Luiz Chaimowicz

**Affiliations:** 1 Department of Computer Science, Federal University of Minas Gerais, Belo Horizonte 31270-901, Brazil; erickson@dcc.ufmg.br (E.R.N.); chaimo@dcc.ufmg.br (L.C.); 2 Department of Automation and Process Integration, Vale Institute of Technology, Ouro Preto 35400-000, Brazil; gustavo.medeiros.freitas@itv.org

**Keywords:** UAV, terrain following flights, optical flow, computer vision, robotics

## Abstract

In this paper, we present a monocular vision-based height estimation algorithm for terrain following flights. The impressive growth of Unmanned Aerial Vehicle (UAV) usage, notably in mapping applications, will soon require the creation of new technologies to enable these systems to better perceive their surroundings. Specifically, we chose to tackle the terrain following problem, as it is still unresolved for consumer available systems. Virtually every mapping aircraft carries a camera; therefore, we chose to exploit this in order to use presently available hardware to extract the height information toward performing terrain following flights. The proposed methodology consists of using optical flow to track features from videos obtained by the UAV, as well as its motion information to estimate the flying height. To determine if the height estimation is reliable, we trained a decision tree that takes the optical flow information as input and classifies whether the output is trustworthy or not. The classifier achieved accuracies of 80% for positives and 90% for negatives, while the height estimation algorithm presented good accuracy.

## 1. Introduction

The use of Unmanned Aerial Vehicles (UAVs) is becoming increasingly popular. Being at a farm or a mine and maybe even at a construction site or for environmental monitoring, UAVs have been extensively used in mapping and surveying applications. Since UAVs use Commercial Off The Shelf (COTS) cameras that are not meant for extreme long-range photography, such vehicles commonly fly at low Above Ground Level (AGL) altitudes. While flying at low altitudes over plane terrain is a simple task that can be easily achieved through the GPS and Inertial Measurement Unit (IMU), flying over rough terrain is considerably more challenging, as it requires the use of specific and expensive sensors, such as rangefinders, or previous knowledge of the flight terrain elevation.

Some applications require low altitude flights, such as mining, mapping and farming, in compliance with aviation regulations. A way of performing such flights is through the use of specific sensors, such as RADAR, SONAR and laser rangefinders; however these are either expensive or provide limited ranges. To avoid using these sensors, a UAV can either use previously known terrain information or fly above the highest point over the desired flight path.

To avoid problems such as ground collisions, typically, when flying a UAV over a mountainous region, the waypoints are determined such that it flies at a constant Above Sea Level (ASL) altitude determined from the highest point on the flight area. However, this could generate suboptimal results depending on the desired mapping application, such as the case of aeromagnetic surveys, where the results are better the closer the vehicle is to the terrain and at a constant AGL altitude. Additionally, the resulting flight plans might be non-compliant with regulatory agencies.

In this paper, we present a height estimation algorithm for terrain following flights based on monocular vision. The algorithm relies on the use of optical flow and projective geometry on a sequence of images for estimating the flight height. A decision tree is used to infer if the results are reliable based on a classification mechanism. Given the associated costs, weight and payload limitations, reliance on public low resolution data, mapping quality and regulations compliance, we decide to extract the maximum amount of information from UAVs’ commonly-used sensors, like images provided from a gimbal-stabilized camera to estimate its Above Ground Level (AGL) altitude. Our approach is designed to work using the hardware that is already available on the UAVs. As they typically carry a stabilized camera to perform mapping tasks, we decided to evaluate the feasibility of a monocular vision-based height estimation algorithm. In order to demonstrate the effectiveness of the proposed approach, both simulations and field experiments were performed.

The paper is organized as follows: Section 2 discusses some related work. In Section 3, we present the adopted methodology, describing in detail the height estimation mechanism and the decision tree used for classification. Section 4 presents the simulations and field experiments performed to evaluate the proposed methodology, while Section 5 brings the conclusions and directions for future work.

## 2. Related Work

This section discusses the inspiration for using optical flow to estimate the distance to objects, which has roots in the field of biology and was later demonstrated in biorobotics experiments, therefore opening the path for using it in field robotics.

### 2.1. Biology and Biorobotics

Horridge [1] conducted the first study that suggests that insects use velocity parallax, similar to optical flow, to control their flight characteristics. He conducted many experiments with mantids to establish a theory of insect vision. He concluded that insects estimate distances to objects based on the apparent difference in velocity between objects and the background; essentially nearer objects move faster than distant ones, a behavior that is irrespective of eye rotation since the eye would have the same angular velocity between objects and the background. The author emphasizes the potential of such visual systems for robotics applications since these insects’ neurons are much slower than electronic components.

In the biorobotics field, researchers have been trying to reproduce insects’ and other animals behavior with robots. A particularly interesting study was first published in 2002 and continues to be developed today [2,3,4,5,6]. To emulate the insects’ eyes, they designed a camera consisting of a 20-pixel linear array and used a plastic aspheric lens at a deliberately defocused focal length to blur the image adjusting the sensor response to their desired parameters. They assembled this camera on an aircraft attached to a mast through an arm, making it free to rotate around the mast at a fixed radius and limited heights to avoid any crashes with surrounding objects. Since the optical flow of the forward region is much lower than the downward, they used a weighted function to compute the optical flow average with greater weights to the frontal pixels. The authors successfully demonstrated that optical flow can be used to allow flying vehicles to perform terrain following flights in a lab-controlled environment.

### 2.2. Robotics

Zufferey et al. [7] uses optical flow sensors to perform collision avoidance of a fixed-wing aircraft flying outdoors through a predefined path using GPS. Their UAV has several computer mice optical flow sensors installed at the tip, placed in an arrow-shaped pattern at different pitch angles, the one at the tip pointing down-most and the farthest ones pointing forward-most, but parallel to the arrow edge. Their technique consists of mapping the optical flow to control signals to perform collision avoidance of the aircraft. During field tests, the vehicle flew at approximately 9 m above the ground and successfully avoided every potential collision with front-lateral and frontal obstacles, for the former performing a slight pitch-up and roll-left maneuver and for the latter a stronger pitch up and slight roll-right maneuver. The authors, however, do not state how the resulting height was verified. In addition, to achieve their goal, they used several low resolution optical flow sensors instead of a single camera that could provide both optical flow data and aerial mapping images.

Herisse et al. [8] uses an optical flow-based terra in following strategy for a Vertical TakeOff and Landing (VTOL) UAV using multiple views. The ork consists of using two cameras, one facing down and one forward, mounted on a quadrotor. While the paper focuses more on the control laws and proofs, the strategy is the same: using optical flow to avoid obstacles. The experimental setup consists of the UAV equipped with two video transmitters that send videos to an off-board computer responsible for calculating the proper control signals and sending them to the vehicle through a radio telemetry link. Since the presented experiments were performed indoors, they used an IMU to estimate the velocity and position of the vehicle, limiting the use of GPS. Therefore, the flight tests were of a short duration due to the increasing errors from integrating these values.

Soccol et al. [9] devises a visual system for optical flow-based guidance of UAVs. An important difference in this approach is the use of a spherical mirror in front of the camera and remapping the image to remove the distortion. This way, what appears below the horizon is at the center of the resulting image, and what appears above the horizon is located at the sides of the remapped image. The authors also remove the rotation-induced optical flow by subtracting the scaled yaw component. The presented experiments consist of using a robotic gantry to move the camera setup in a lab-controlled environment and have shown that after remapping and removing the rotational optical flow, the computations are quite accurate. We believe that image remapping and using scaled rotational flow corrections could benefit the further development of this work.

The reader is referred to Chao et al. [10] for a complete survey of optical flow techniques for robotics navigation applications.

#### Height Estimation

One of the most thorough works on height estimation presented by Moe [11] is based on passive aircraft altitude estimation using computer vision. The goal is to use passive sensing instead of the commonly-used radartechnology to estimate the vehicle’s height, reducing the chance of military aircraft being detected. According to the author, laser, RADAR or ultrasound rangefinders are quickly ruled out because they are active sensors. The remaining alternatives are out-of-focus blur, stereo vision and structure from motion. The author highlights the ability to choose the interval between the frames in optical flow estimation based on aircraft altitude and motion and the use of camera motion information to improve the optical flow computation.

Some possible advantages of optical flow are: a dense grid of height estimates, capability to detect obstacles or moving objects and a constant amount of computation needed for each frame. Moe points out that errors could be induced due to wrong aircraft motion estimation. While the translational error is linearly dependent on the translation estimation error, the rotational error is non-linear, and a $0.03^{\circ }$ error would already be accountable. One possibility to reduce this error consists of maximizing the translation between subsequent images, which can be done by pointing the camera straight down. On the other hand, this would not allow the aircraft to detect obstacles far ahead. Therefore, the camera is positioned facing forward, with a small tilt down.

To reduce the error, the author introduces a temporal filtering mechanism, which consists of selecting the appropriate frame interval depending on the aircraft motion and altitude. Two types of filtering are considered: weighted least squares and a Kalman filter. The former is used for optical flow due to its lower computational cost and the latter for region tracking, due to its better accuracy and smaller entries.

In a simple initial experiment, seven images were used, and the error was below 1%. The real flight experiments used four different pieces of footage, from a small archipelago, above a church, at the runway of an airport and, lastly, a maneuver sequence. In the archipelago sequence, the aircraft flew at an approximately 820-m altitude, and the optical flow deviations from RADAR were around ±10 m, while the region tracking was approximately ±5 m. On the church sequences, the errors were greater (around 10%) due to the ground not being plane. On the runway sequence, the errors were around 2%, again indicating that it works more accurately over plane terrain. The last sequence, with maneuvers, has errors of up to 30%, which indicates that the technique is not suitable for high rotations.

Garratt and Chahl [12] conducted field experiments of a UAV terrain following flight using optical flow. They used a Yamaha RMAX helicopter carrying a camera, a laser range finder to be used as the ground truth and a differential GPS unit to account for the camera translation. Their algorithm, however, does not account for the vehicle’s vertical motion. In their experiments, the flight was conducted over flat terrain, and the AGL altitude was only 2 m. With this setup, the error was around 10%. This prevented the analysis of the algorithm behavior at higher altitudes and for different terrain, such as a mountain.

Differently from these approaches, our approach consists of developing a methodology to perform height estimation, specifically designed for terrain following flights. Other contributions are estimation reliability classification through decision trees and the consideration of the vehicle vertical displacement in the optical flow computation. Given the adoption of COTS hardware, we had to use a gimbal-stabilized camera, since the image sensor features a running shutter, which would make it impossible to digitally stabilize the image or use it to estimate the translational optical flow through de-rotation techniques.

## 3. Methodology

**Figure 1:**
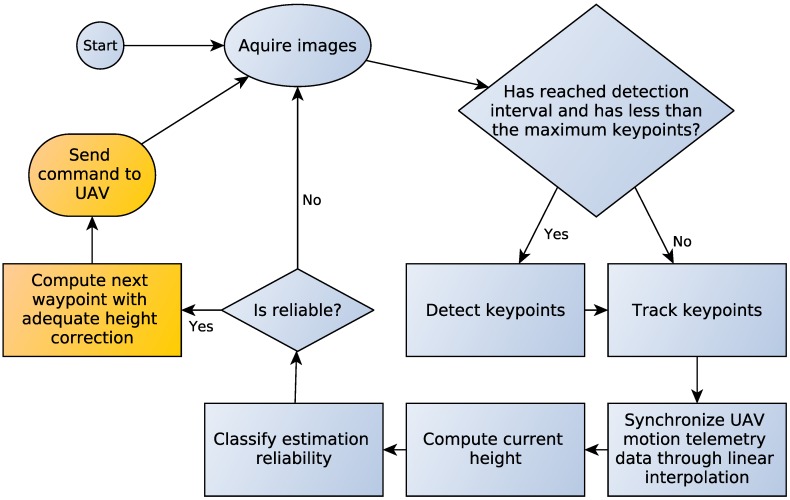
Height estimation methodology. The system takes images as input and detects the Shi and Tomasi [14] keypoints at predefined intervals. Then, it tracks the keypoints through Lucas and Kanade [13] optical flow, takes the flow vectors and synchronizes the image information with the UAV telemetry data in order to compute the aircraft height. Finally, the system verifies if the estimation is accurate through a decision tree classification. The yellow boxes are further improvements currently being developed and are not part of this work.

The proposed estimation algorithm computes the height through optical flow together with motion information provided by the UAV’s flight controller after GPS and IMU data fusion. To estimate the UAV’s height, our approach was inspired by the classical stereo vision technique, however utilizing images provided from a single camera acquired at different times. An overview with the main steps of our methodology is shown in Figure 1. The following assumptions are considered:

- the scene is mostly static;
- the camera translation is approximately known;
- there is no independent camera rotation.

### 3.1. Keypoint Detection and Tracking

Our methodology consists of using the sparse Lucas and Kanade optical flow [13] to track keypoints proposed by Shi and Tomasi [14] from videos obtained by UAV, as well as its motion information to estimate the flying height. First, a set of pixels is selected by the Shi and Tomasi method, which computes for each pixel its corner quality measure and filters the low quality corners out. Second, every pixel presenting a high quality corner is tracked by assuming that the flow field is smooth locally. Such an assumption allows us to use the approach of least square solving proposed by Lucas and Kanade. It is worth noting that, since Shi and Tomasi’s detector selects pixels presenting large variance in their neighborhood (large eigenvalues), the linear system of Lucas and Kanade’s approach will give a well-conditioned matrix providing high quality tracking. This tracking step provides the motion of the selected pixels, which are used to estimate the height by triangulation.

### 3.2. Height Estimation

Using the tracked keypoints and vehicle motion computed from the velocities provided by the UAV’s IMU properly synchronized with the video through linear interpolation, we calculate the vehicle’s height.

**Figure 2:**
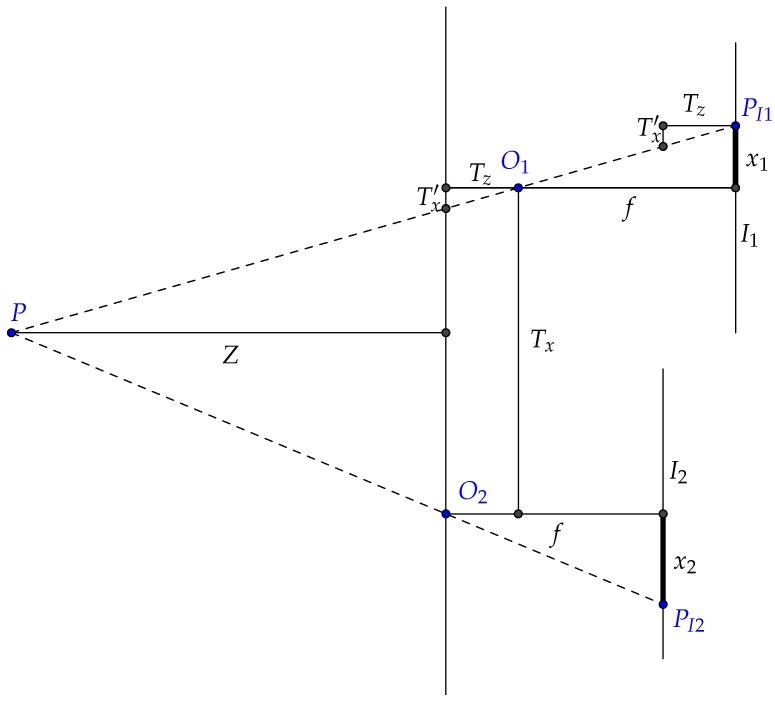
Adapted stereo schematics for 2D motion. The figure illustrates a point *P* projected on two different images, denoted by points $P_{I1}$ and $P_{I2}$. *Z* stands for the depth, *f* for the focal length, $T_{x}$ and $T_{z}$ for translations, $x_{1}$ and $x_{2}$ the distances from the projected points to the principal point and $T_{x}^{\prime }$ a projected compliment of the translation along the *x* axis.

Figure 2 shows a 2D scenario of the height estimation technique. Let *P* be a 3D point on the scene projected on two different images (i.e., frames). The points $O_{1}$ and $O_{2}$ indicate the focal points from each acquisition. Note that both images are acquired at different depths. The 2D points $P_{I1}$ and $P_{I2}$ indicate the projected points at the image plains ($I_{1}$ and $I_{2}$).

The value of depth *Z* is given by:

(1) $$Z=\frac{fT_{x}-T_{z}x_{1}}{x_{1}+x_{2}},$$

where *f* is the focal length, $T_{x}$ and $T_{z}$ are the translations in the *x* and *z* axes, $x_{1}$ and $x_{2}$ are the distances from the projected points to the principal point and $T_{x}^{\prime }$ to a projected compliment of the translation along the *x* axis, which is computed as:

(2) $$T_{x}^{\prime }=T_{z}\frac{x_{1}}{f}.$$

One can see that Equation (1) is similar to the classic stereo equation, with the exception of the term $T_{z}x_{1}$, which is responsible for adjusting the depth estimation with relation to the camera translation along the *z* axis.

**Figure 3:**
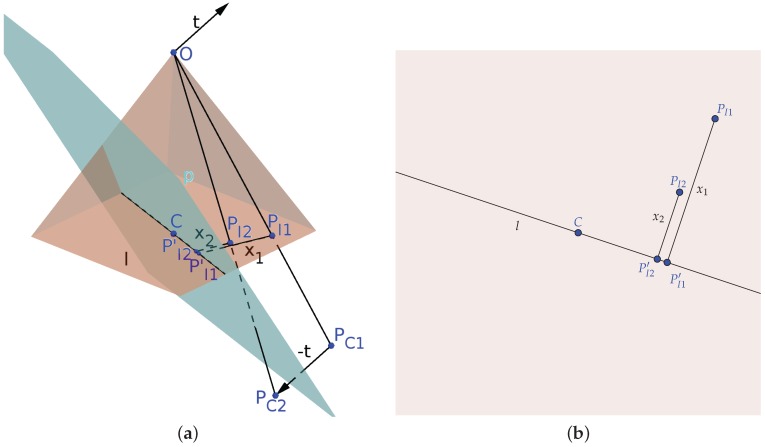
Adapted stereo schematics for 3D motion. (**a**) The projection of a point $P_{W}$ in two consecutive images after a camera motion represented by $\vec{t}$. For viewing purposes we aligned both camera frames and displaced the point $P_{W}$ by $-\vec{t}$, represented in the camera coordinate system as points $P_{C1}$ and $P_{C2}$ for the first and second frame, respectively; these points are then projected onto the image plane as points $P_{I1}$ and $P_{I2}$; (**b**) the image plane. (**a**) Projection schematic; (**b**) image plane.

Figure 3 shows how to compute the depth in a 3D translation scenario. To properly estimate the depth, we project a plane *p*, which is perpendicular to $\vec{t}$ and passes through the camera principal point *C*. Finally, we compute the distances from $P_{I1}$ and $P_{I2}$ to the line *l* intersecting *p* and the image plane.

It is important to note how the translation along the *z* axis is represented. In this case, we consider a negative translation when the UAV moves up and positive when it moves down, so the coordinates are aligned with the camera coordinate system. A change in this representation would imply a change of the signs of the equation.

To estimate the depth, we then use Equation (1) with the computed values, taking into account on which side of line *l* the points $P_{I1}$ and $P_{I2}$ lie, changing the sign accordingly. To verify that, we compute:

(3) $$S=sign(\left(\vec{P_{I2}^{\prime }P_{I1}^{\prime }}\times \vec{CP_{I1}}\right)\cdot \left(\vec{P_{I2}^{\prime }P_{I1}^{\prime }}\times \vec{CP_{I2}}\right)).$$

Additionally, by combining Equation (3) with Equation (1), we get:

(4) $$Z=\frac{fT_{xy}-T_{z}x_{1}S}{x_{1}+x_{2}S},$$

where $T_{xy}$ is the horizontal movement of the camera and $T_{z}$ the vertical one, such that $T_{xy}+T_{z}=\vec{t}$.

### 3.3. Reliability Classification

It is worth noting that there are situations where the height estimation based on optical flow becomes inaccurate, such as when there is very small motion between the frames or when there is too much yaw. Therefore, we decided to create an additional fail-safe by training a decision tree to determine whether the estimations are reliable or not and only use the results classified as reliable.

Considering specific situations where our height estimation would be inaccurate, e.g., large rotations or low UAV velocity, we use a classifier to determine when its use is reliable. The classifier is based on decision trees, more specifically the ID3 (Iterative Dichotomiser 3) [15], because they are fast and can be easily implemented on embedded hardware.

We create feature vectors composed of the average and standard deviations of the optical flow vectors’ magnitudes and directions. To avoid discontinuities when the direction changed from $0^{\circ }$ to $359^{\circ }$, we used the sine function instead of the vector’s raw orientation. To train the decision tree, we assign classes to data according to how close they are to the ground truth and manually label good or bad frames according to our perception. The tree is then used to classify in which frames the height estimation is reliable.

### 3.4. Time Complexity

The time complexity of the whole procedure is O(nlogn) when using the median height and O(n) for average height. It is important to note that the most demanding task is the decoding of the video files, which is made without hardware acceleration in our experiments. However, when working directly with the video input devices, the algorithm performs much faster, with real-time performance.

The keypoint detection is linear in the number of pixels examined in the image, since we detect the set of keypoints using the Shi and Tomasi approach, which computes the corner quality for each pixel by the trace and the determinant of a symmetric matrix composed of the neighborhood of a fixed size around each pixel. The tracking and motion estimation are linear in the number of keypoints extracted by Shi and Tomasi. The amount of computation needed to solve the normal equation of least squares is constant because the neighborhood is fixed. The height estimation is O(nlogn) when using the median height. After computing the height for each keypoint, it is linear in the number of keypoints, because the number of operations of the triangulation computed by Equation (4) is constant for each keypoint; we look for the median height by sorting the list of height. This cost decreases to O(n) when using the average height. The reliability classification is linear in the number of keypoints, because the classification is performed by decision trees, which have the time complexity of the order O(h), where *h* is the height of our tree, which is fixed after the training.

## 4. Experiments and Results

This section describes the experiments and discusses the results. The experiments consist of evaluation through simulations, as well as several field experiments consisting of flights in different areas that comprise diverse terrain and relief types.

In order to estimate the height, we evaluated three different metrics: 2D mean, 3D mean and 3D median. The former, used by Garratt and Chahl [12], is an approach that disregards the vertical displacement of the camera and estimates the height based on the global average of the depth computed for each tracked point. The second, likewise, still uses the global average, but accounts for the vertical movement of the vehicle. The latter also considers the vertical motion, but instead of the average of the computed depths, it uses the median. Furthermore, as the 3D median performed better than the others, we chose to omit them from the presented graphics for a cleaner visualization of the data; however, we still provide a quantitative analysis between all metrics at the end of this section.

The 2D mean consists of estimating the height using only the horizontal displacement of the vehicle in addition to the video, being less accurate in situations that involve vertical motion of the aircraft, which is typically a consequence of scenarios requiring terrain following flights.

The 3D mean, based on our methodology, properly accounts for the vertical motion of the UAV; thus, it is a better model for the height estimation problem; however, since it depends on estimating the traveling direction of the aircraft to determine the line l, from Figure 3b, it is more susceptible to noise. It results in a more accurate, however less precise height estimation.

The 3D median applies the same principle as the 3D mean, but instead of computing the height as the average of the estimations, it takes the median of them, therefore filtering erroneous estimations caused by a few high disturbing values, presenting more accuracy and precision than the previous one.

### 4.1. Simulations

To set most of the input parameters in the experiments and evaluate the proposed methodology at a controlled environment, we first performed several test using a realistic simulation scenario. For this, we use FlightGear [16], as it is highly integrated with ground elevation databases, as well as the very same GCS (Mission Planner [17]) used in our field experiments.

**Figure 4:**
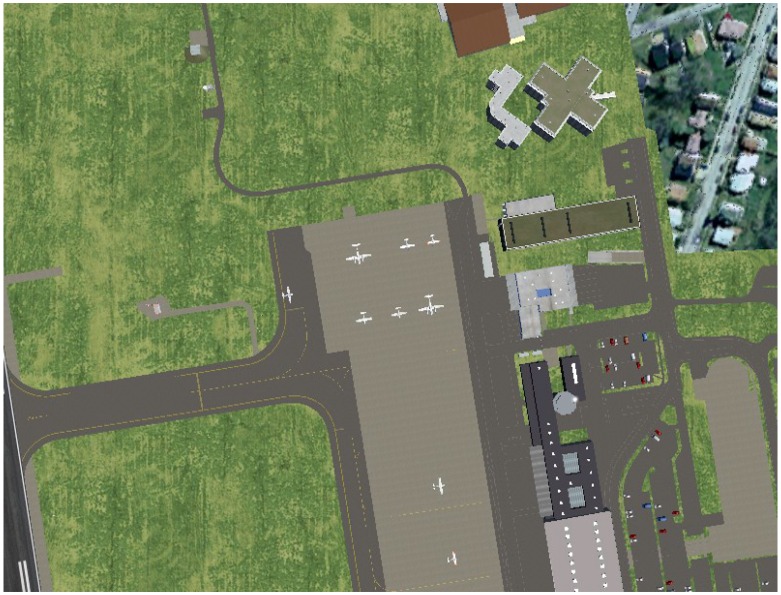
Screenshot of a simulation at the Innsbruck airport using the FlightGear Flight Simulator.

Furthermore, the FlightGear Flight Simulator presents the advantage of using 3D models, satellite imagery and custom textures to render a scenario based on a mesh created from the elevation databases. Furthermore, it is open source, which allows changes in several parameters. For example, it is possible to set the camera positioning, simulating the behavior of biaxial or triaxial gimbals. A sample picture is shown in Figure 4.

We combined FlightGear with the ArduCopter [18] SITL (Software In The Loop) simulator, which was responsible for computing the vehicle motion and control, sending these data to the simulator for rendering and also logging data in the same format our vehicle would log in the real world. In fact, it is the same software that runs on our UAV.

We performed the simulations at the Innsbruck airport, which, according to the FlightGear wiki, is one of the places with the best scenery data available. Moreover, it also presents the necessary slopes to properly investigate the feasibility of our height estimation approach for terrain following flights. We performed two flights, one at a fixed ASL altitude of 500 m above the runway and the other at a fixed AGL altitude of 200 m.

#### 4.1.1. Fixed ASL Altitude at 500 m above Runway

**Figure 5:**
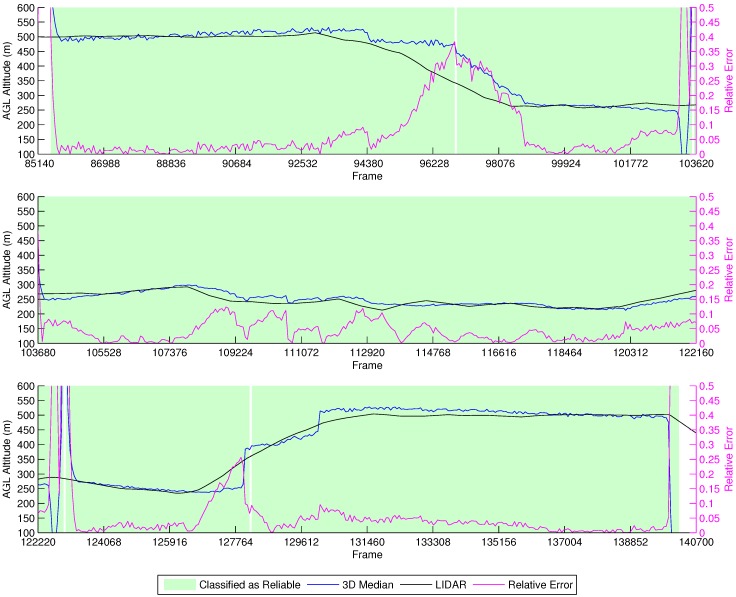
Height estimation results for the fixed ASL altitude at 500 m above the runway simulation.

Figure 5 shows the results of the height estimation for this simulation. The *x* axis indicates the frame in the video file; the left *y* axis shows the AGL altitude of the vehicle; and at the secondary *y* axis, the relative estimation error of the current frame is presented.

It is possible to observe that, in most of the frames, the estimations are close to the actual AGL altitude of the vehicle, but when the terrain starts to rise, reducing the effective height, we observe a delay for the estimations to reach the actual height of the vehicle. The reason for this behavior is because at 500 m, the field of view comprises so much area that many optical flow vectors are accumulated at the part of the frame opposite the vehicle movement. Indeed, this is confirmed by the fact that the delay decreases proportionally with the AGL altitude. However, when the AGL altitude starts to rise again, we see two sudden spikes at the estimations. This is due to the fact that the simulations rely on synthetic textures, and the keypoint detector might identify more representative corners in some textures than others, causing sudden changes in the tracked features when they go out of view.

To allow the classifier to determine if the estimations were reliable, we trained it for such heights. However, we did not mix data from the simulations at the training of the field experiments’ decision tree, which will be discussed in the next sections. There are two points, close to Frames 103,435 and 102,775, that present some spikes in the height estimation values. These are points where the vehicle stops and changes direction and are classified as not reliable by our decision tree (the frames in green on the graph are classified as reliable). This behavior is also observed elsewhere in our experiments.

#### 4.1.2. Fixed AGL Altitude at 200 m

**Figure 6:**
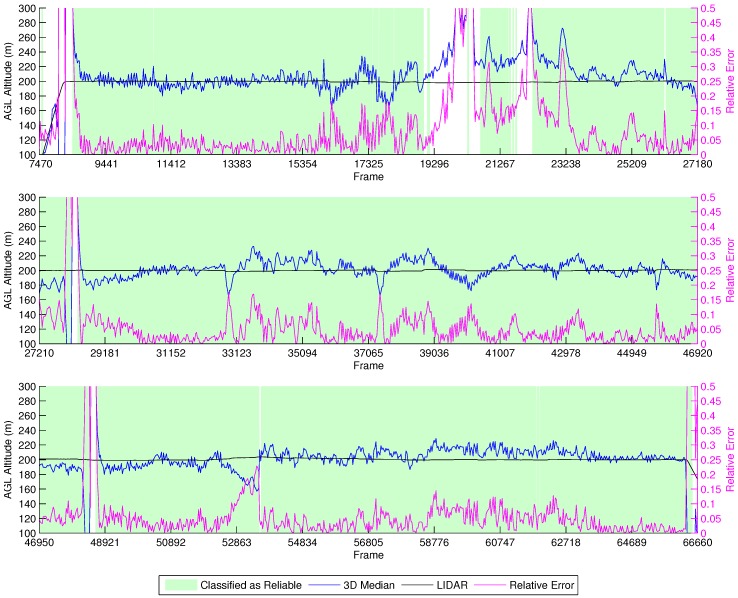
Height estimation results for the fixed AGL altitude at 200 m simulation.

Figure 6 depicts the simulation results for the fixed AGL altitude at 200 m experiment. One can clearly observe that the data have more jitter in this simulation, which implies that the tracked points are being exchanged more frequently, a consequence of flying lower than the previous simulation. However, the great majority of estimations are still within 10% of the actual height. Around Frames 19,296–21,750, the vehicle flies over a river, which presents reflections, therefore compromising the estimations. This effect was minimized in the previous simulation because the area being captured by the camera was larger. We also observe two spikes when the vehicle stops and changes directions, at Frames 28,195 and 48,430, a characteristic also shown in the previous simulation at the same locations.

### 4.2. Field Experiments

To test the height estimation algorithm with different terrain inclinations, we performed two field experiments: flying over a small hill and over a plane with low inclination and few obstructions.

**Figure 7:**
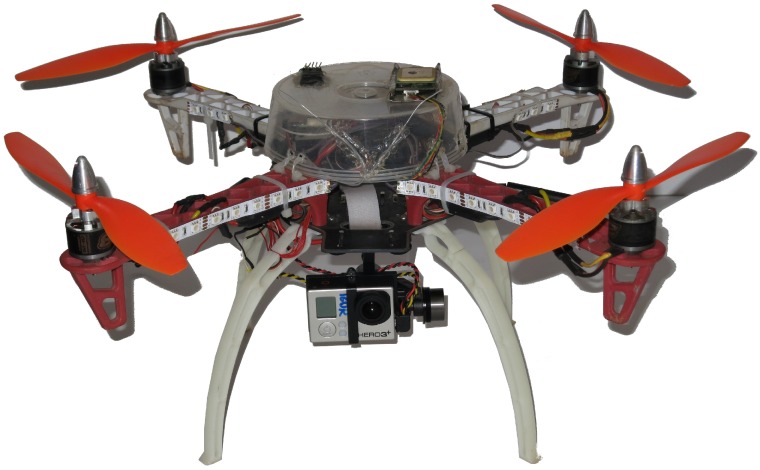
The UAV prototype we used on our experiments.

To perform the experiments and evaluate the proposed methodology, we designed and constructed a UAV. The objective was to build a low-cost vehicle, easy to assemble and composed of COTS parts. Furthermore, the UAV should be capable of performing autonomous missions, preferably using open source software for better support. We decided to use a multirotor platform because it has Vertical TakeOff and Landing (VTOL) capability, allowing operations in rough terrain, is simpler to operate and maintain, while also being safer than traditional helicopters. As we were targeting a low-cost vehicle, we chose a quadrotor platform, as it uses fewer motors, therefore being the least expensive one. Figure 7 shows the final prototype. The vehicle cost ended up being less than 500 USD, with potential for being even less in present day, as many of the components’ prices have decreased.

#### 4.2.1. Hill

**Figure 8:**
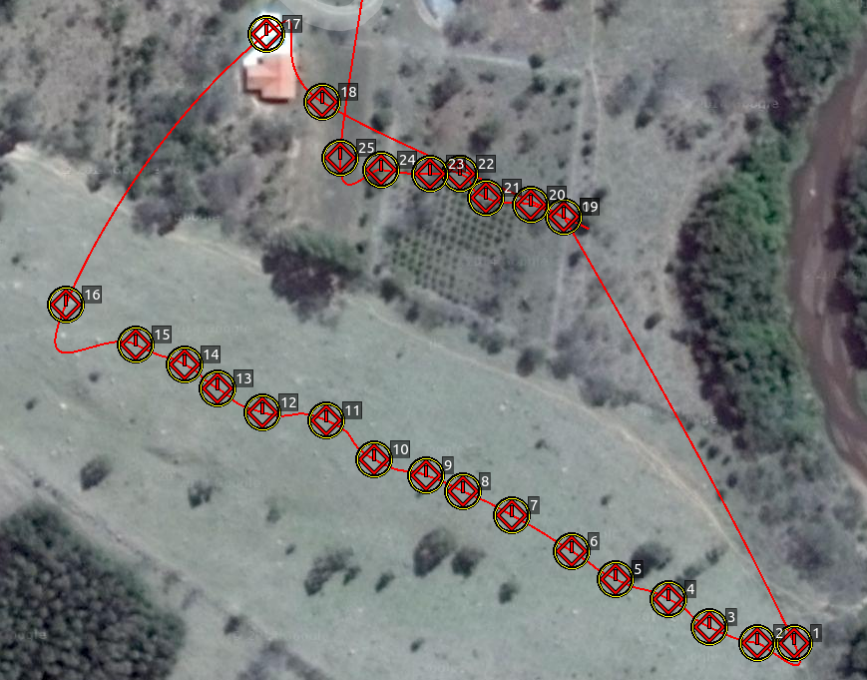
Hill flight plan. The figure shows the flight plan for the hill video sequence: the first slope is between Waypoints 1 and 16 and the second between Waypoints 19 and 25.

**Figure 9:**
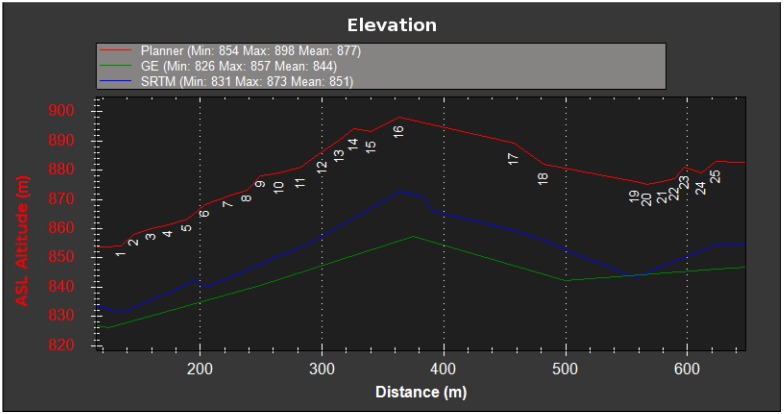
Hill flight elevation. The figure shows the elevation at the specified coordinates provided by the flight plan (25 m above ground), Google Earth (GE) and the Shuttle RADAR Topography Mission (SRTM) databases. This graph was generated with the Mission Planner Ground Control Station (GCS).

**Figure 10:**
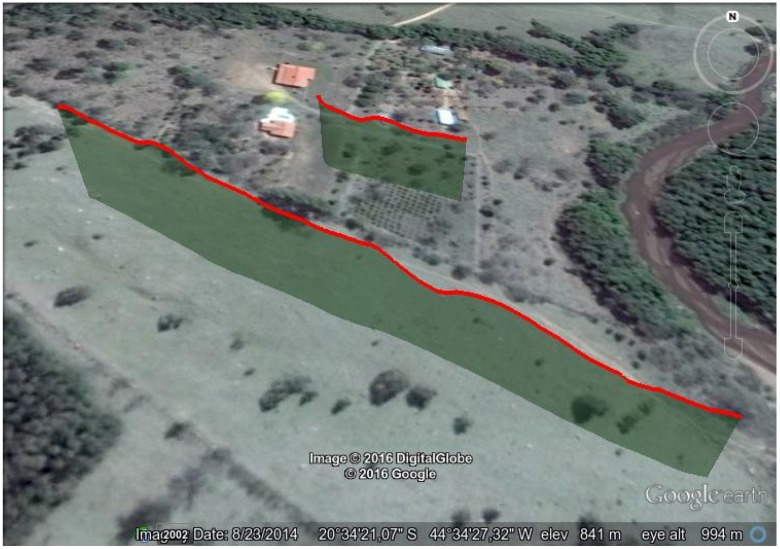
Hill flight path from GPS data of the telemetry logs. This image is from a perspective view, where the red lines represent the flight paths of the two areas of interest and the green shade the projection of this flight path on the Earth’s surface.

**Figure 11:**
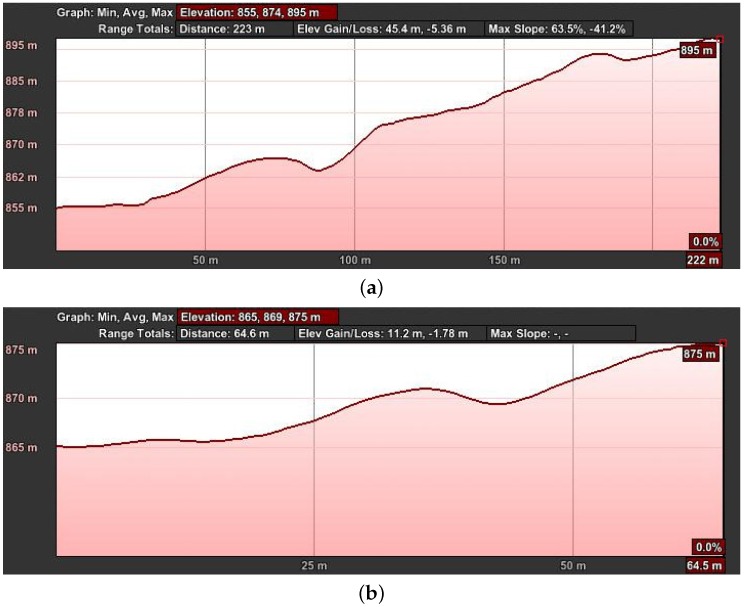
Hill flight path elevation from GPS data of the telemetry logs. The horizontal axis shows the distance the UAV has traveled, and the vertical axis represents the ASL altitude. These graphs were generated using Google Earth. (**a**) First part; (**b**) second part.

This first experiment consists of flying over two different parts of a small hill, one mostly covered by grass and the other by small trees and bushes. The vehicle had a GoPro camera configured to record 1920×1080 video at 60 FPS with automatic white balance and a narrow FOV. Figure 8, Figure 9, Figure 10 and Figure 11 show the flight plan and telemetry data of the experiment.

In this experiment, we established a ground truth by logging the projection on the terrain of the desired flight path through a mobile phone GPS application and adjusting the waypoints accordingly to keep the UAV at a fixed AGL altitude of 25 m.

**Figure 12:**
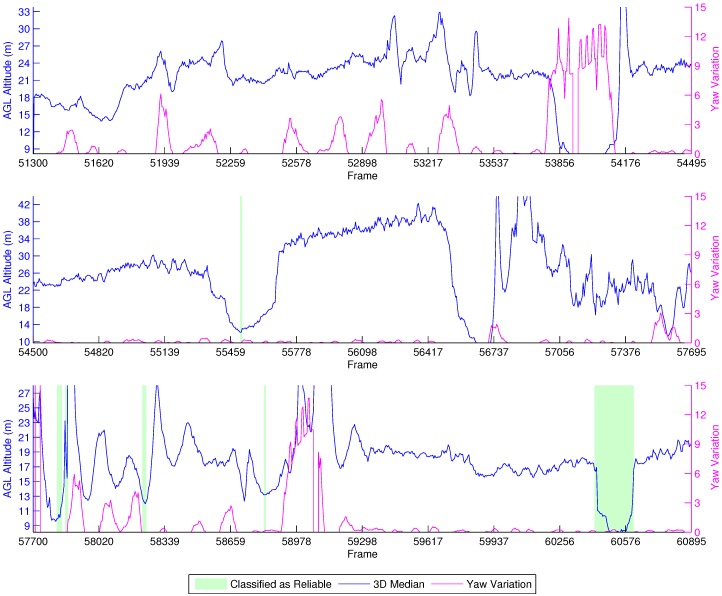
Height estimation results for the farm experiment.

Figure 12 shows the results of this experiment. The secondary *y*-axis represents the absolute yaw variation of the vehicle through the last five frames instead of the relative error, as the LIDAR-lite was unavailable at the time. Moreover, using just the GPS altitude from the flight log (25 m) would result in noisy information itself, being unsuitable for a proper analysis of the errors. Notwithstanding, the experiment still produces valuable data that confirm the high impact of yaw in the estimations. It can be observed that the yaw interferes with the height estimation, as the estimations are much more stable with no yaw.

The beginning of the experiment shows the vehicle flying over a small tree around Frames 51,300–51,779. Then, it flies uphill with some yaw interference up to Frame 53,379, and after that, it flies straight to the next waypoint, at Frame 53,857. Continuing on, the UAV rotates to face the next waypoint at Frame 56,500, and in the meantime, we see the AGL altitude increasing as this part of the flight actually consists of going at a fixed ASL altitude; and the terrain altitude is decreasing. This behavior changes only around Frames 55,457–55,617 as the vehicle flies over a tree. Upon reaching the next uphill sequence, the aircraft rotates to face the following waypoint, at Frame 58,900. This time, the altitude estimates oscillate more because of the bushes and trees below, as the terrain is full of orange trees. After that, the UAV flies at a fixed ASL altitude to the next waypoint, and this is again shown by the height variation.

Unfortunately, our reliability classifier was trained with data from real flights executed at an AGL below 12 m. Because of this, most of the estimations were classified as not reliable, since the decision tree was not trained to correctly classify images acquired from such heights. Around Frames 60,417–60,600, the vehicle flies closely over a tree, and the height estimation drops below 11 m. In this situation, we observe that the classifier identifies these as reliable estimations.

#### 4.2.2. Plane Terrain

The second set of field experiments was performed at the Federal University of Minas Gerais (UFMG) campus, where there is a clear field with a small inclination and few obstructions. These experiments were performed using a GoPro camera configured to record 1920×1080 video at 60 FPS, a narrow FOV and automatic white balance.

**Figure 13:**
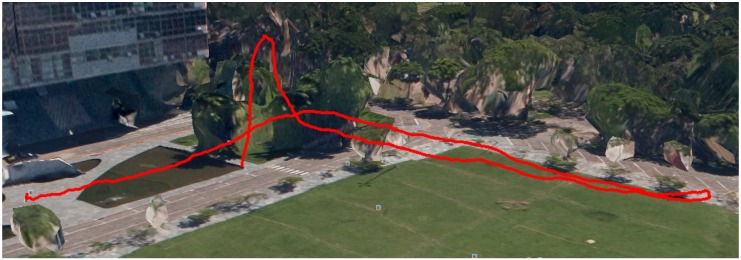
Autonomous flight path.

**Figure 14:**
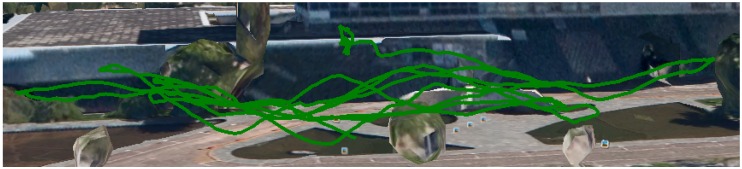
Manual flight path with vertical zig-zag pattern.

**Figure 15:**
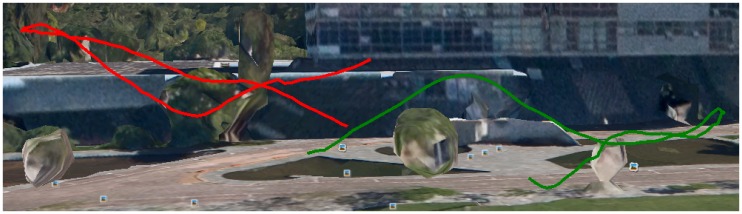
Selected sequences of the manual flights for clear visualization.

We performed different flights: an autonomous flight (Figure 13) at fixed ASL altitude and also manually-controlled flights (Figure 14 and Figure 15) performing a vertical zig-zag pattern to test the algorithm response to height changes. We chose to perform the latter manually as we required drastic changes in the vertical speed of the vehicle quite close to the ground, and an autonomous flight could result in a collision. The main disadvantage of manual controlled flights is that the horizontal speed is not as constant as for the autonomous ones.

To establish a reliable ground truth, we installed a LIDAR-Litelaser rangefinder to the gimbal, alongside the camera, ensuring that it is always pointing to the center of the captured frames. This LIDAR has an accuracy of 2.5 cm at a maximum distance of 50 m. The sensor values are stored in a flight log that can be used to replay the mission or can be instantaneously read though the serial telemetry link by the ground station computer.

After synchronizing the data, we could integrate the vehicle’s velocities from the flight logs to determine its displacement between frames and easily compare the data from our height estimation with the LIDAR-Lite data.

##### Autonomous Flight at Fixed above Sea Level (ASL) Altitude

**Figure 16:**
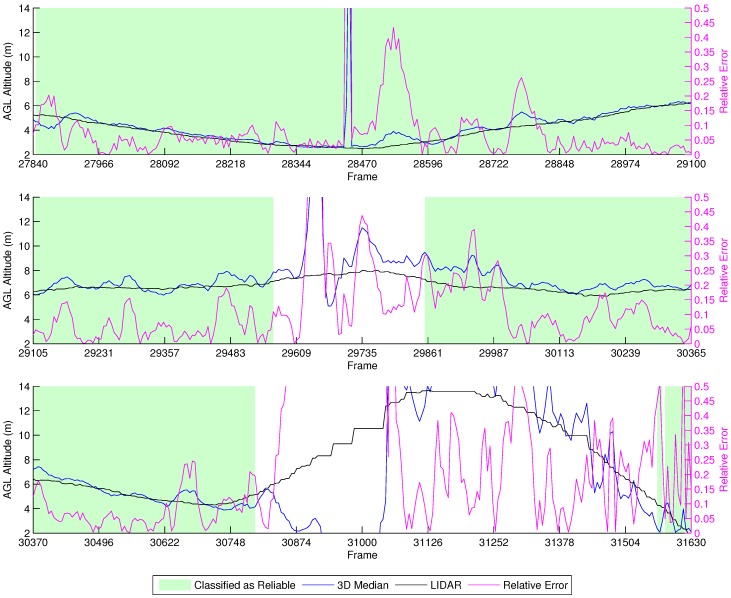
Height estimation results for the LIDAR experiment at a fixed ASL altitude.

Figure 16 shows the results of our fixed ASL altitude flight. From the beginning up to Frame 27,840, the vehicle takes off, but it is unstable. After flying towards the first waypoint, the classifier marks the estimation as reliable, and all of the algorithms closely match the LIDAR data until the vehicle stops at Frame 29,570 and reverses direction, going back to the previous waypoint before performing a Return To Launch (RTL) maneuver at Frame 30,800. The RTL maneuver is when the vehicle autonomously flies to the point from which it took off. However, as a safety measure, the vehicle first climbs to a safe height predefined at 15 m, before going to the landing position, which can be observed in the last part of the graph.

This experiment shows that there is a very distinct reaction in the height estimation when the vehicle is stopping and reverting direction, which can be seen between Frames 29,591 and 29,757. The classifier is capable of identifying this behavior, marking the estimations from this period as unreliable. Furthermore, the estimations from the RTL maneuver are also marked as unreliable.

##### Manual Vertical Zig-Zag

**Figure 17:**
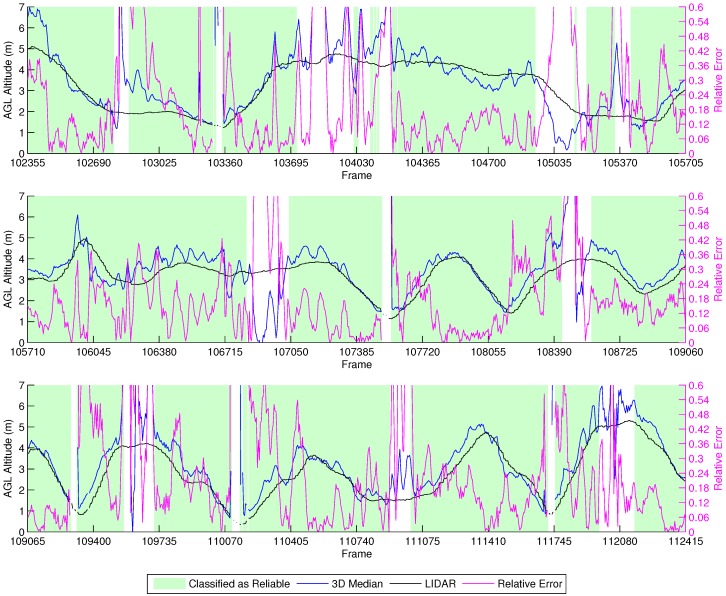
Height estimation results for the manual vertical zig-zag experiment.

Figure 17 shows the results from the manual vertical zig-zag flights. Due to the tight error margins of performing this experiment without a high accuracy localization device, we decided to perform it manually, thus preventing collisions of the vehicle with the ground. We observe that the height estimation performed by our methodology is close to the LIDAR. Furthermore, the characteristic up/down spikes we noticed in the previous experiments when the vehicle stops and changes direction were correctly labeled by our classifier as unreliable. Thus, the height estimations in this experiment provided good results in comparison to the LIDAR data when the classifier identified them as reliable.

#### 4.2.3. Quantitative Analysis

After performing the visual analysis of the graphs, we proceeded to the quantitative analysis, in which we performed tests to evaluate the algorithm performance with the LIDAR experiments’ data. The results are presented in Table 1.

To achieve these results, we used only the data classified as good by our decision tree. The tree achieved accuracies of 80% for true positives and 90% for true negatives. According to our labels, a positive result means our estimative was reliable. The decision tree highly improves the accuracy and precision of our height estimation, and it is fundamental for safety reasons.

First, we analyzed the algorithm accuracy and precision through the average and standard deviation of the relative height estimation errors. However, it became clear that despite our efforts to filter bad estimations through our classification technique, few disturbing points remained, resulting in some alterations of the averages and standard deviations. Therefore, to filter these data, we limited the relative errors to up to 250%, discarding higher errors. To verify if we were discarding a considerable amount of data, we counted the used samples for each metric. The counts revealed that our 3D median algorithm is using almost as much data as the traditional 2D mean approach, while our 3D mean algorithm is just a bit behind.

We observed that the results from the 3D mean algorithm are worse than the others. This is because, as already mentioned, our methodology is more susceptible to noise from poorly-tracked features. On the other hand, the 3D median algorithm leads to better accuracies (indicated by the averages) than the traditional 2D mean algorithm for the manual flight experiment, which, unlike the fixed ASL altitude, had vertical motion. The achieved accuracies were of 17.1% versus 19.9% for the manual experiment that featured vertical motion.

Results from the manual flights are worse than the autonomous ones due to large variations in the speed and motion of the vehicle. The more constant the motion of the vehicle is, the more stable the estimations are. Unfortunately, a quadrotor platform is normally easy to use, but greatly unstable.

We also computed common error metrics, the Mean Squared Error (MSE) and the Mean Absolute Error (MAE), this time without the additional filtering limiting the relative errors to 2.5. Once again, the 3D mean algorithm produced worse results and 2D mean, and 3D median algorithm behaved similarly, with a slight advantage to the former.

## 5. Conclusions

This work presents a monocular vision-based height estimation algorithm for terrain following flights. The algorithm tracks features between consecutive images computing the optical flow and uses projective geometry to estimate the height. Since some of these estimations can be unreliable, especially when the vehicle reverts its flying direction or performs sharp maneuvers, a decision tree is used to infer if the results are reliable based on a classification mechanism. The proposed methodology was evaluated both in simulations and real experiments, and the results were satisfactory, showing that optical flow and monocular vision can be used in real-world scenarios to estimate an aircraft AGL altitude.

The quantitative data reveal that the algorithm performs well with sufficient motion and show the importance of flying at a constant speed, but it still needs to be further improved towards increased precision. The achieved accuracy, on the other hand, was demonstrated to be greater than comparable techniques, such as the traditional 2D approach. We are confident that using optical flow de-rotation will increase its robustness to yaw, therefore increasing the precision.

In summary, we showed the feasibility of using monocular vision to enable UAVs to perform terrain following flights and plan on continuing to develop this research to integrate the estimations in a waypoint generator to control the vehicle’s AGL altitude. We also plan on continue working in this research towards a real-time onboard height estimation and control approach for UAVs, preferably discarding the necessity of a gimbal in the future through the use of global shutter cameras attached to IMUs. Furthermore, we would also like to experiment with similar techniques for collision avoidance.

## Figures and Tables

**Table 1:** Quantitative results of the classifier filtered data. The first row represents the experiments; the first column the metrics used; the second row contains the average height, in meters, computed exclusively with the LIDAR (LIght Detecting and Ranging) data, of each experiment; the second column contains the evaluated algorithms. In addition to the classifier filter, for the averages and standard deviations, we filtered the errors up to 250% and represented the results in relative numbers. The MSE (Mean Squared Error) and MAE (Mean Absolute Error) are based on absolute numbers, filtered only by the classifier.

	Experiment		Autonomous Fixed ASL Altitude	Manual Vertical Zig-Zag
Metric		Average Height (m)	4.800	2.648
	Algorithm			
Average	2D Mean		0.051	0.199
	3D Mean		0.135	0.200
	3D Median		0.070	0.171
Standard Deviation	2D Mean		0.169	0.384
	3D Mean		0.343	0.389
	3D Median		0.226	0.366
Count	2D Mean		555	2460
	3D Mean		541	2400
	3D Median		554	2447
MSE	2D Mean		0.370	4.148
	3D Mean		13.469	70.519
	3D Median		1.507	5.398
MAE	2D Mean		0.388	0.964
	3D Mean		0.984	1.578
	3D Median		0.395	1.010

## Abbreviations

The following abbreviations are used in this manuscript:

UAV	Unmanned Aerial Vehicle
GPS	Global Positioning System
COTS	Commercial Of The Shelf
AGL	Above Ground Level
IMU	Inertial Measurement Unit
RADAR	RAdio Detecting And Ranging
SONAR	SOund Navigating And Ranging
ASL	Above Sea Level
VTOL	Vertical TakeOff and Landing
LK	Lucas and Kanade
ID3	Iterative Dichotomiser 3
GCS	Ground Control Station
SITL	Software In The Loop
LIDAR	LIght Detecting And Ranging
USD	United States Dollar
FPS	Frames Per Second
FOV	Field Of View
GE	Google Earth
SRTM	Shuttle Radar Topography Mission
RTL	Return To Launch
MSE	Mean Squared Error
MAE	Mean Absolute Error

## References

[1] Horridge G.A. (1986). A Theory of Insect Vision: Velocity Parallax. Proc. R. Soc. Lond. B Biol. Sci..

[2] Netter T., Francheschini N. A Robotic Aircraft that Follows Terrain Using a Neuromorphic Eye. Proceedings of the IEEE/RSJ International Conference on, Intelligent Robots and Systems.

[3] Ruffier F., Franceschini N. Visually Guided Micro-Aerial Vehicle: Automatic Take off, Terrain Following, Landing and Wind Reaction. Proceedings of the ICRA ’04 2004 IEEE International Conference on Robotics and Automation.

[4] Ruffier F., Franceschini N. (2005). Optic flow regulation: The key to aircraft automatic guidance. Robot. Auton. Syst..

[5] Franceschini N., Ruffier F., Serres J., Viollet S. (2009). Optic Flow Based Visual Guidance: From Flying Insects to Miniature Aerial Vehicles. Aerial Vehicles.

[6] Expert F., Ruffier F. (2015). Flying over uneven moving terrain based on optic-flow cues without any need for reference frames or accelerometers. Bioinspir. Biomim..

[7] Zufferey J.C., Beyeler A., Floreano D. Autonomous Flight at Low Altitude with Vision-Based Collision Avoidance and GPS-Based Path Following. Proceedings of the 2010 IEEE International Conference on Robotics and Automation (ICRA).

[8] Herisse B., Oustrieres S., Hamel T., Mahony R., Russotto F.X. A General Optical Flow Based Terrain-Following Strategy for a VTOL UAV Using Multiple Views. Proceedings of the 2010 IEEE International Conference on Robotics and Automation (ICRA).

[9] Soccol D., Thurrowgood S., Srinivasan M. A Vision System for Optic-Flow-Based Guidance of UAVs. Proceedings of the Australasian Conference on Robotics and Automation.

[10] Chao H., Gu Y., Napolitano M. A Survey of Optical Flow Techniques for UAV Navigation Applications. Proceedings of the 2013 International Conference on Unmanned Aircraft Systems (ICUAS).

[11] Moe A. (2000). Passive Aircraft Altitude Estimation Using Computer Vision. Licentiate Thesis.

[12] Garratt M.A., Chahl J.S. (2008). Vision-based terrain following for an unmanned rotorcraft. J. Field Robot..

[13] Lucas B.D., Kanade T. (1981). An Iterative Image Registration Technique with an Application to Stereo Vision. Proceedings of the 7th International Joint Conference on Artificial Intelligence—Volume 2, Vancouver, BC, Canada, 24–28 August 1981.

[14] Shi J., Tomasi C. Good Features to Track. Proceedings of the CVPR ’94 1994 IEEE Computer Society Conference on Computer Vision and Pattern Recognition.

[15] Quinlan J. (1986). Induction of decision trees. Mach. Learn..

[16] FlightGear Flight Simulator—Sophisticated, Professional, Open-Source. http://www.flightgear.org/.

[17] Mission Planner Overview—Mission Planner Documentation. http://ardupilot.org/planner/docs/mission-planner-overview.html.

[18] Copter Home—Copter Documentation. http://ardupilot.org/copter/index.html.

